# Pathological Study of Rare Malignant Cardiac Tumors: A Case Series of Five Patients

**DOI:** 10.1155/crip/8876442

**Published:** 2026-02-13

**Authors:** Mahshid Hesami, Kambiz Mozaffari, Behnaz Jahanbin

**Affiliations:** ^1^ Cardiovascular Research Center, Rajaie Cardiovascular Institute, Tehran, Iran; ^2^ Department of Pathology, Cancer Institute, Imam Khomeini Hospital Complex, Tehran University of Medical Sciences, Tehran, Iran, tums.ac.ir

**Keywords:** case reports, histopathology, Iran, malignant cardiac tumor, rare tumors

## Abstract

Malignant cardiac tumors are uncommon but highly aggressive growths that may develop within the heart or spread from other primary locations. Considering the significance of pathological studies and the dissemination of findings on malignant cardiac tumors in advancing medical knowledge and informing future research in this complex area, this study is aimed at presenting cases of malignant cardiac tumors identified in patients treated at the Rajaie Hospital, Tehran, Iran. In this study, we focused on five cases of malignant cardiac tumors documented in the hospital′s surgical pathology archives spanning from 2020 to 2024. The identified tumors consisted of invasive thymoma, diffuse large B‐cell lymphoma, primary cardiac synovial sarcoma, primary undifferentiated pleomorphic sarcoma, and metastatic renal cell carcinoma, all classified as rare cardiac neoplasms.

## 1. Introduction

Cardiac tumors encompass benign congenital tumors, benign acquired tumors, and malignant tumors (MTs). Although cardiac tumors are rare, they rank among the most common metastatic tumors [1] and can be categorized into primary and secondary. Primary cardiac tumors are notably less frequent than secondary malignant lesions, occurring in approximately 0.001%–0.3% of autopsies. The majority of surgically removed primary cardiac tumors are benign, accounting for almost 90% of cases. Until recently, cardiac myxomas were widely recognized as the most prevalent benign cardiac neoplasm in adulthood, constituting nearly 80% of benign tumors. Conversely, secondary malignant diseases affecting the heart and pericardium are substantially more common than primary cardiac malignant diseases, estimated to be 30–1000 times more prevalent [2].

In a random autopsy study, metastatic involvement was identified in 0.4% of cases, while in patients with confirmed cancer, cardiac involvement can be as high as 20%. Tumors typically spread to the heart through direct tumor extension, venous/lymphatic spread, or arterial metastasis. The most common underlying malignant diseases associated with secondary cardiac involvement include carcinoma of the lung, breast, esophageal, gastric, renal, melanoma, lymphoma, and leukemia [1].

The primary malignant cardiac tumors exhibit varying clinical characteristics, which are influenced by factors like the tumor′s location, size, invasiveness, friability, and rate of growth [3–5]. The majority of these tumors are sarcomas. Sarcomas originating in the heart tend to progress rapidly and can result in early death due to infiltration of the myocardium, obstruction of circulation, or the spread of metastases to the lungs, lymph nodes, and liver [6]. Surgical intervention is the preferred treatment option whenever feasible, although recurrence is common, and survival is typically limited to 1 year. However, complete resection has shown to be associated with long‐term survival, particularly in cases of low‐grade sarcomas. On the other hand, primary cardiac lymphomas present with symptoms such as cardiac tamponade, atrial fibrillation, right heart failure, and superior vena cava (SVC) syndrome. Cardiac lymphomas generally have a more favorable prognosis than sarcomas, with approximately 40% of patients achieving a complete response to systemic therapy. Therefore, when a cardiac tumor is diagnosed, establishing a comprehensive differential diagnosis is particularly challenging because of the rarity and diverse presentation of these tumors [3, 5].

In Iran, few studies have explored cardiac metastases and their association with the primary neoplasms that infiltrate the cardiovascular system. Anvari et al. demonstrated that the prevalence of metastatic cardiac tumors is more than primary ones, and breast cancer is the prevalent cause of metastasis. Also, the symptoms presented by patients could vary significantly based on the tumor′s location within the heart [7]. Previously, rare cardiac MTs have been reported, including metastatic renal cell carcinoma (RCC) to the inferior vena cava and the right atrium (RA), SVC syndrome, undifferentiated/unclassified cardiac sarcoma, and undifferentiated pleomorphic sarcoma [2, 6, 8–11]. Given the importance of pathological studies and the publication of findings on malignant cardiac tumors for advancing medical knowledge, improving patient care, and guiding future research in this challenging field, this case study is aimed at reporting on rare malignant cardiac tumors observed in patients. A pathological evaluation was conducted on a series of five cases involving malignant cardiac tumors that underwent surgical intervention at Rajaie Hospital.

## 2. Materials and Methods

### 2.1. Case Selection and Clinical Information

This study was conducted in accordance with the ethical guidelines of Rajaie Hospital Center and received approval from the medical committee of the Rajaie Cardiovascular Research Institute (Tehran, Iran). Over the past 5 years (2020–2024), 152 cardiac mass samples were submitted to the pathology department. Among these, 110 were diagnosed as myxomas, 26 as benign cardiac tumors other than myxomas, and 16 as MTs. The MTs included angiosarcoma (three cases), leiomyosarcoma, intimal sarcoma (two cases), rhabdomyosarcoma, pleomorphic undifferentiated sarcoma, synovial sarcoma, lymphoma (two cases), invasive thymoma, and metastases originating from cervical carcinoma, papillary thyroid carcinoma, colon carcinoma, and renal carcinoma. For this study, we focused on five cases of malignant cardiac tumors documented in the hospital′s surgical pathology archives spanning from 2020 to 2024. It is worth noting that the other rare MTs mentioned above (diagnosed in this period) have either been published previously or are in the process of publication (see References [2, 6, 12]). Clinical data were extracted from medical records and included patient age, gender, tumor size, site of occurrence, and clinical symptoms.

### 2.2. Special Staining and Immunohistochemistry

All tumor sections stained with hematoxylin and eosin (H&E) were examined under a light microscope. Immunohistochemical analysis was conducted on paraffin‐embedded tissue using antibodies directed against various antigens, with negative and positive controls routinely performed.

## 3. Results

### 3.1. Clinical Findings

Essential information of all cases is summarized in Table 1.

**Table 1:** Clinical and pathological information of five patients diagnosed with rare malignant cardiac tumors. Gender: female (F) and male (M). NA, no information available.

Case no.	Age (y)	Sex	Tumor size (cm)	Main symptoms	Location	Status	Type of resection
1	54	F	4 × 3 × 1.5	Weight loss and weakness	Left atrium	Metastatic renal cell carcinoma	Tumor debulking
2	32	M	6 × 5.5 × 1.5	Palpitation, dyspnea, and syncope	Left atrium	Primary undifferentiated pleomorphic sarcoma	Tumor debulking
3	60	M	4.5 × 3 × 24.5 × 4 × 2.5	NA	‐ Pericardium‐ Right atrium	Primary cardiac biphasic synovial sarcoma	Complete resection
4	60	M	10 × 9 × 4	Syncope and weight loss	Right atrium	Diffuse large B‐cell lymphoma	Complete resection
5	70	F	5 × 3.5 × 28 × 5 × 25 × 6 × 3	Vena cava syndrome	‐ Anterior mediastinum‐ Right atrium‐ Superior vena cava	Invasive thymoma	Complete resection

### 3.2. Pathological Findings (Microscopy)

#### 3.2.1. Metastatic RCC

**Figure 1:**
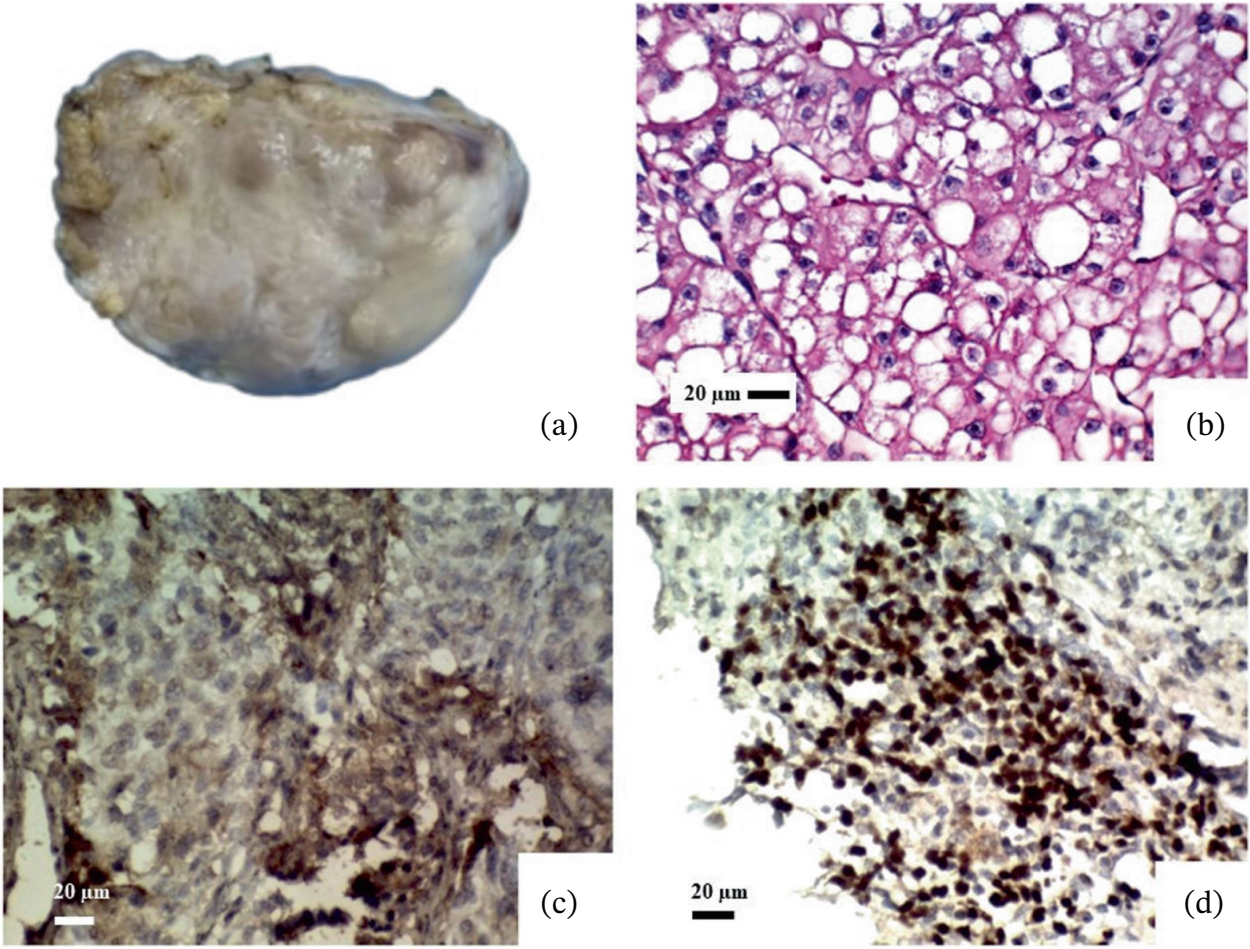
Metastatic renal cell carcinoma. (a) An ovoid cream‐colored mass with soft consistency. (b) Microscopic examination (H&E stain) reveals nests and sheets of epithelial cells with clear to eosinophilic cytoplasm, distinct membranes, and mildly enlarged nuclei. (c) Immunohistochemical studies show a positive cytoplasmic reaction for CD10. (d) Positive nuclear reaction for PAX8 (all objective magnification: 40X).

A 54‐year‐old woman underwent a medical workup due to weight loss and weakness and was diagnosed with RCC (core needle biopsy) and left atrial mass. The patient underwent left radical nephrectomy and LA mass excision. Gross examination of the kidney reveals one round, brown‐colored mass in the upper lobe measuring 2 cm in diameter. Cardiac mass shows one piece of ovoid cream tissue with soft consistency measuring 4 × 3 × 1.5 cm. Microscopic examination of renal and cardiac masses reveals that neoplastic tissue contains nests and sheets of epithelial cells with clear to eosinophilic cytoplasm, with distinct membranes and mildly enlarged nuclei, some with eosinophilic nucleoli. An IHC study of cardiac mass reveals a positive cytoplasmic reaction of tumoral cells for CD10 and a positive nuclear reaction for PAX8 (Table 2 and Figure 1).

**Table 2:** Differential diagnoses based on immunohistochemistry.

Tumor	Positive	Negative
Metastatic renal cell carcinoma	CD10 and PAX8	—
Primary undifferentiated pleomorphic sarcoma	CD68 and Ki‐67	DESMIN, C‐kit, and ALK
Primary cardiac synovial sarcoma	CKAE1/AE3, TLE1, BCL2, and DESMIN	STAT6
Diffuse large B‐cell lymphoma	LCA, CD20, Ki‐67, and CD3	CK, CD30, S100, CD1A, CD10, and BCL6
Invasive thymoma	—	—

### 3.3. Primary Undifferentiated Pleomorphic Sarcoma

**Figure 2:**
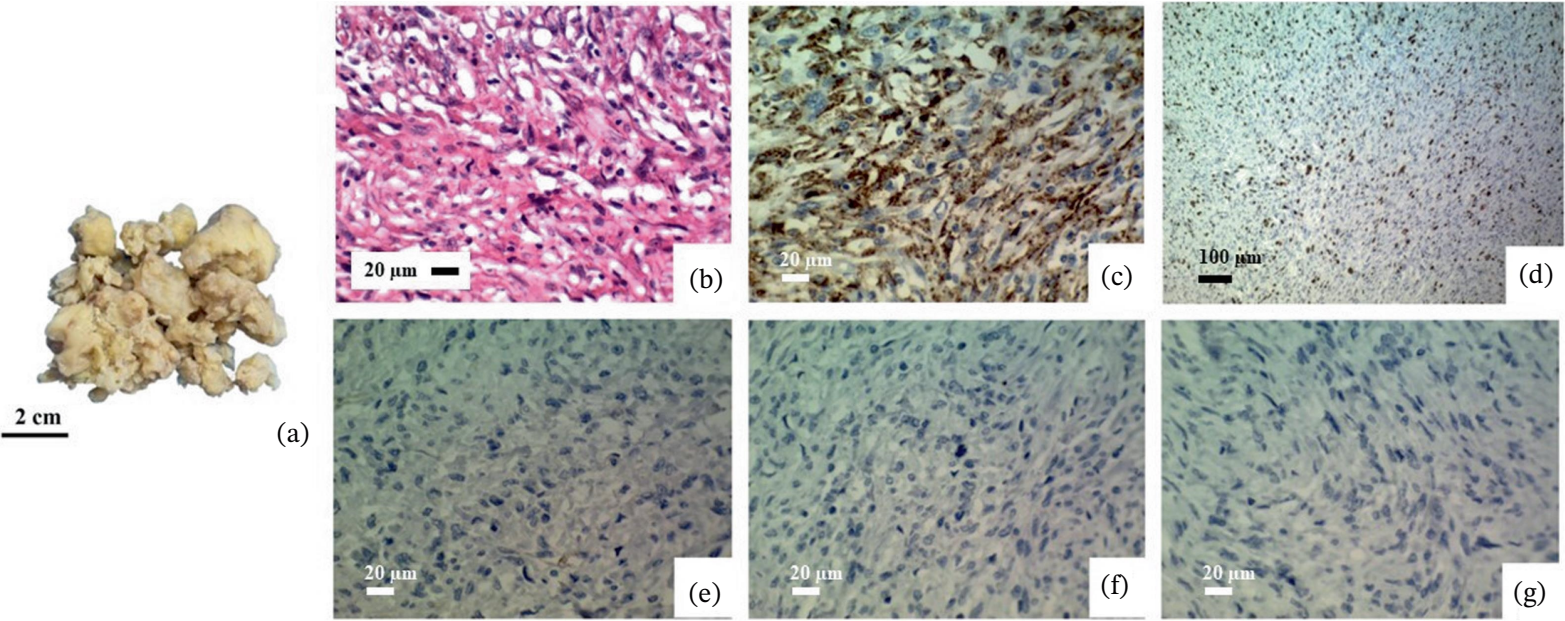
Primary undifferentiated pleomorphic sarcoma. (a) Gross examination of the left atrial mass shows multiple pieces of cream tissue with soft and fragile consistency. (b) Microscopic examination (H&E stain) reveals that neoplastic tissue contains fascicles of atypical spindle cells and some epithelioid cells with enlarged vesicular nuclei, moderate nuclear pleomorphism, and prominent nucleoli with eosinophilic cytoplasm. (c, d) Immunohistochemical studies show positive cytoplasmic staining for CD68 and Ki‐67 (with a proliferation index ranging between 60% and 70%). (e–g) Negative reaction for DESMIN, C‐kit, and ALK1 (objective magnification of b, c, and e–g: 40X, d: 10X).

A 32‐year‐old man presented with palpitations and dyspnea 1 month ago and was diagnosed with a left atrial mass, then underwent LA mass debulking. Gross examination of the left atrial mass shows multiple pieces of cream tissue with soft and fragile consistency, totally measuring 6 × 5.5 × 1.5 cm. Microscopic examination reveals that neoplastic tissue contains fascicles of atypical spindle cells and some epithelioid cells with enlarged vesicular nuclei, moderate nuclear pleomorphism, and prominent nucleoli with eosinophilic cytoplasm. Frequent mitoses (about 40 per 10 high‐power fields [HPFs]) and multiple foci of necrosis are seen. The IHC study shows a positive reaction for CD68 (most of the cells) and a negative reaction for DESMIN, C‐kit, and ALK1. The Ki‐67 proliferation index ranged between 60% and 70% (Table 2 and Figure 2).

### 3.4. Primary Cardiac Biphasic Synovial Sarcoma

**Figure 3:**
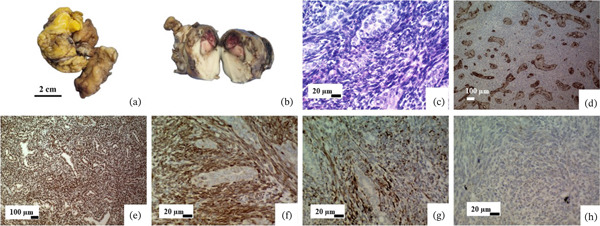
Primary biphasic synovial sarcoma. (a) The pericardial mass consists of two pieces of creamy yellowish tissue with fatty consistency, including one cream‐colored mass. (b) The right atrial mass shows two pieces of lobulated cream‐colored tissue with foci of necrosis. (c) Microscopic examination of two tumors reveals a biphasic synovial sarcoma composed of fascicles of atypical spindle cells and epithelioid component with granular and nested structures. (d–g) IHC supports diagnosis through positive reaction for CKAE1/AE3, TLE1, BCL2, and DESMIN (patchy positive). (h) Negative reaction for STAT6 (objective magnification of c and f–h: 40X, d and e: 10X).

A 60‐year‐old man presented with right atrial and pericardial masses. Gross examination of the pericardial mass shows two pieces of creamy yellowish tissue with fatty consistency measuring 5 × 2.5 cm, including one creamy ovoid mass measuring 4.5 × 3 × 2 cm. The right atrial mass shows two pieces of lobulated cream tissue measuring 4.5 × 4 × 2.5 cm with foci of necrosis. Based on histomorphological findings and IHC results, pericardial and right atrial masses showed similar features. Both tumors consist of fascicles of spindle cells with plump hyperchromatic nuclei, inconspicuous nucleoli, and indistinct cytoplasm with areas of glandular structures lined by cuboidal cells and small solid nests of epithelioid cells with distinct amphophilic cytoplasm with round to ovoid nuclei. The mitotic count was 12 per 10 HPFs. The IHC study shows a positive reaction for CKAE1/AE3, TLE1, BCL2, and DESMIN (patchy positive) and a negative reaction for STAT6 (Table 2 and Figure 3).

### 3.5. Diffuse Large B‐Cell Lymphoma

**Figure 4:**
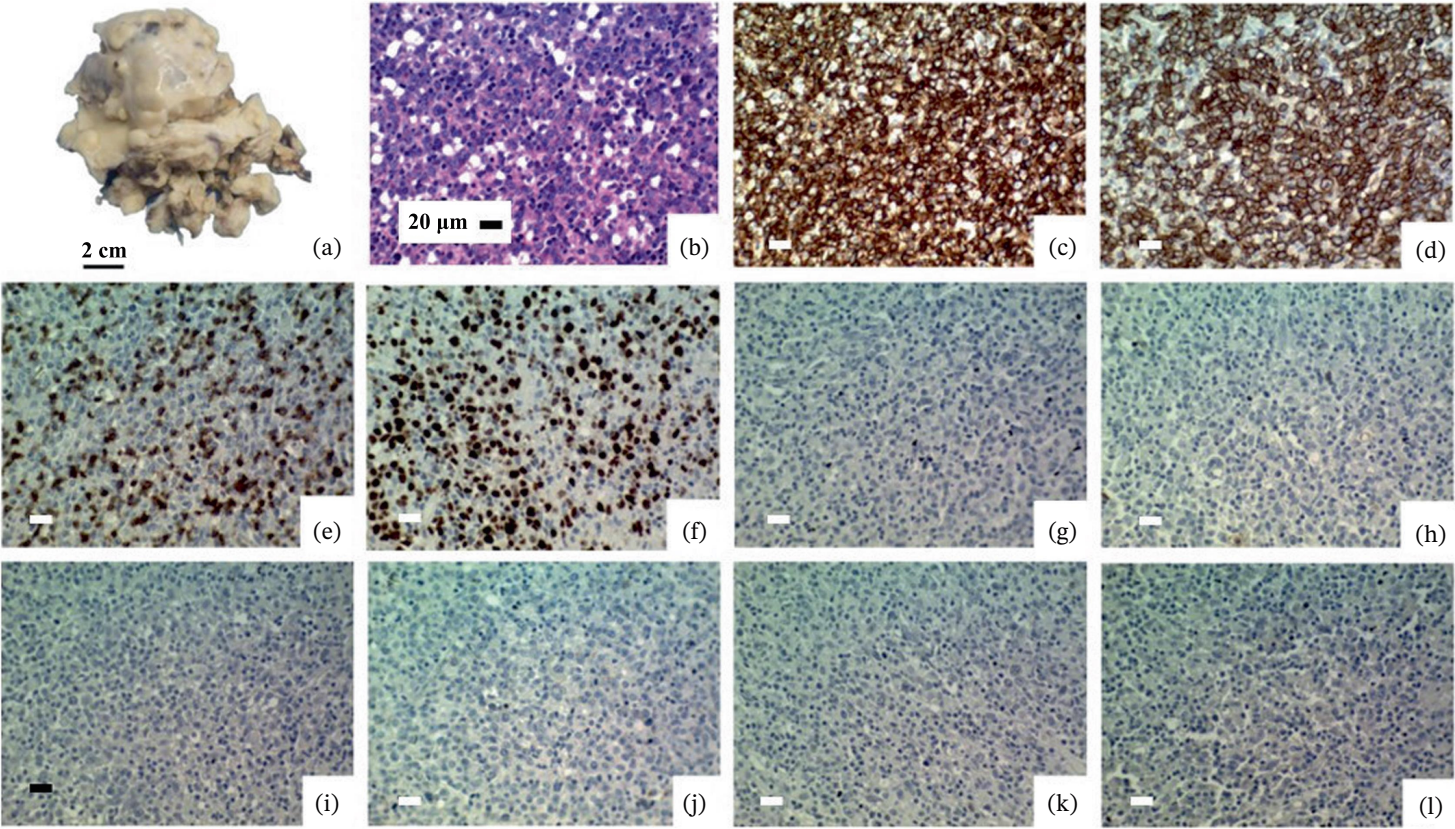
Diffuse large B‐cell lymphoma. (a) Gross examination shows multiple irregularly shaped fragments of creamy yellowish tissue. (b) Microscopic examination shows cardiac myocytes infiltrated by a neoplasm with a diffuse pattern of large atypical cells exhibiting pleomorphic vesicular nuclei and prominent nucleoli. (c) IHC shows staining for LCA. (d) CD20 is positive in large cells. (e) CD3 is positive in small background lymphocytes. (f) Ki‐67 proliferation index is 40%. (g–l) Negative staining is observed for CD30, CD1A, S100, BCL6, CD10, and CKAE1/AE3 (all scale: 20 *μ*m, all objective magnification: 40X).

A 60‐year‐old man presented with syncope and weight loss (within the last month) and multiple right atrial masses. Gross examination of the right atrial mass shows multiple pieces of creamy yellowish, irregularly shaped tissue, totally measuring 10 × 9 × 4 cm. Microscopic examination shows cardiac myocytes infiltrated by neoplasm with a diffuse pattern of large‐sized round cells with vesicular nuclei, prominent nucleoli, frequent mitoses, and necrosis. The IHC study reveals a positive reaction for LCA, CD20 (in large cells), and CD3 (in small background lymphocytes) and a negative reaction for CKAE1/AE3, CD30, CD1A, S100, BCL6, and CD10. The Ki‐67 proliferation index is 40% (Table 2 and Figure 4).

### 3.6. Invasive Thymoma

**Figure 5:**
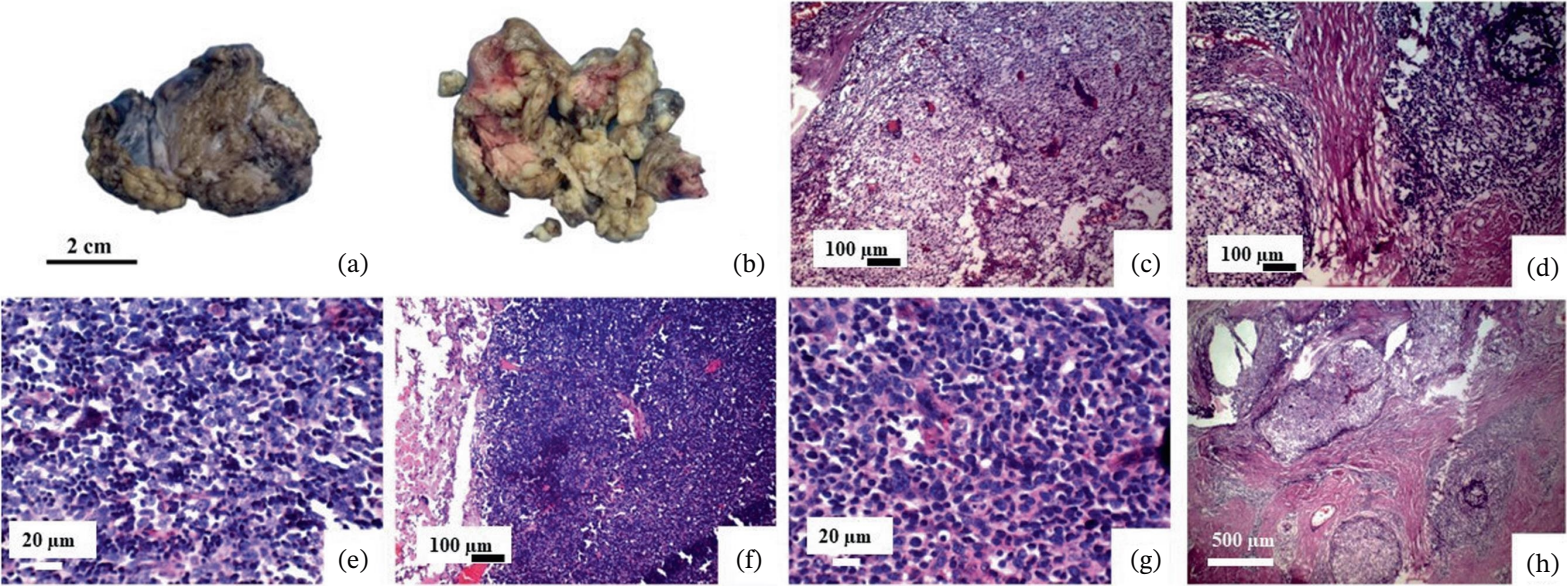
Invasive thymoma. (a, b) Anterior mediastinal and right atrial masses consist of pieces of creamy brownish, irregularly shaped tissue with creamy whitish cut surfaces. (c, d, h) Mediastinal mass sections show neoplastic lobules of large polygonal epithelial cells with vesicular nuclei and clear cytoplasm, admixed with a few lymphocytes. (e–g) The right atrial and SVC masses display neoplastic lobules intersected by fibrous bands, containing scattered polygonal epithelial cells with vesicular nuclei, prominent nucleoli, and moderate pleomorphism in a lymphocytic‐rich background (objective magnification of c, d, and f: 10X; e and g: 40X; h: 4X).

A 70‐year‐old woman presented with SVC syndrome with anterior mediastinal (A), right atrial (B), and SVC masses (C). (A) The mediastinal mass consists of one piece of creamy brownish, irregularly shaped tissue measuring 5 × 3.5 × 2 cm with creamy whitish cut surfaces. (B) The right atrial mass consists of multiple pieces of creamy brownish tissue measuring 8 × 5 × 2 cm. (C) The SVC mass consists of one piece of tube‐like tissue measuring 5 × 6 × 3 cm (Figure 5).

(A) Sections show that neoplastic tissue contains cellular lobules intersected by a fibrous band. Some lobules consist of large polygonal epithelial cells with round to ovoid vesicular nuclei and clear cytoplasm, admixed with a few small lymphocytes. Some lobules show scattered large polygonal epithelial cells with round vesicular nuclei and clear cytoplasm in a lymphocytic‐rich background (mixed Type B2 and B3). (B and C) Neoplastic tissue contains cellular lobules intersected by a fibrous band. Lobules reveal scattered polygonal epithelial cells with round to ovoid vesicular nuclei, prominent nucleoli, and moderate nuclear pleomorphism in a lymphocytic‐rich background. Frequent mitoses, necrosis, and vascular spaces with peripheral palisading of tumoral cells were seen (Type B2).

## 4. Discussion

Patients diagnosed with cardiac tumors typically present with nonspecific symptoms that vary based on the tumor′s location and the degree of invasion into adjacent tissues. Therefore, cardiac tumors must undergo histological analysis to verify the diagnosis and exclude the possibility of malignancy, thereby facilitating the optimal treatment plan. Also, currently available cardiac imaging methods do not offer a conclusive diagnosis [13]. The infrequency of malignant primary cardiac tumors, coupled with their diverse clinical manifestations, often leads to incidental diagnoses of intracardiac masses. Malignant cardiac tumors are rare but highly aggressive neoplasms that can originate in the heart or metastasize from other primary sites. The most common types of primary malignant cardiac tumors include angiosarcomas, rhabdomyosarcomas, undifferentiated sarcomas, leiomyosarcomas, and fibrosarcomas, each with distinct histological features and clinical presentations [14, 15]. The prognosis for these sarcomas is often poor due to their rapid growth and tendency to invade the myocardium.

Although cardiac metastases are often clinically silent, they should always be considered in any individual with new cardiac symptoms and known malignancy. Metastases can reach the heart (frequently originating from melanomas, lung cancer, or breast cancer) via lymphatic or hematogenous pathways, or through direct or transvenous extension [16]. Lymphatic spread generally results in pericardial metastases, while hematogenous spread is more likely to cause myocardial metastases, leading to a complex clinical scenario [17–19]. Additionally, extracardiac tumors may extend into the atria and heart chambers through transvenous spread. For instance, RCC has been reported to grow intraluminally through the renal vein and inferior vena cava, reaching the RA in about 1% of cases [17]. Cardiac metastases of RCC are rare and are often challenging to manage. In our patient, the presence of tumor cells in the kidney with similar morphology, along with positive nuclear staining for PAX8 and cytoplasmic staining for CD10 (Figure 1), supports the diagnosis of RCC over other types of clear cell carcinoma [20]. RCC is a highly aggressive form of cancer, representing 3% of all malignancies in humans, and is recognized as the deadliest among urologic cancers. Herein, we present a case of LA metastasis from RCC in a patient (Table 1 and Figure 1). There is no established standard management for cardiac metastases from RCC. Most reported cases of cardiac metastases from RCC have been managed surgically to decline in quality of life resulting from heart failure [21–23]. For unrespectable tumors, systemic therapies like tyrosine kinase inhibitors have shown some tumor regression and symptom relief [16, 22–24]. Metastasis of RCC to the left atrium is exceptionally rare; this case underscores the importance of considering metastatic RCC in the differential diagnosis of a left atrial mass in a patient with a history of renal cancer. Most patients with RCC metastases are asymptomatic and may present with various symptoms. Hypertension is observed in 20%–38% of cases, while other cardiac symptoms include syncope, dyspnea, chest pain, cough, and peripheral edema. Cardiac tumors may compress or block coronary arteries, potentially causing myocardial infarction or heart failure. Pericardial involvement with effusion and cardiac tamponade often leads to hemodynamic compromise [22, 24].

Invasive thymoma has the potential to infiltrate the pleura, pericardium, and various mediastinal structures [25]. The WHO classifies thymomas into two main types based on the shape of the neoplastic epithelial cells and their nuclei: Those with spindle or oval‐shaped cells are classified as Type A, while those with dendritic or plump (epithelioid) cells are classified as Type B. Tumors that exhibit both morphologies are categorized as Type AB. Type B thymomas are further divided into three subtypes, based on the relative increase in neoplastic epithelial cells (in comparison to lymphocytes) and the degree of cellular atypia [26]. SVC syndrome linked to thymomas is uncommon, occurring in approximately 4% of cases. The first documented case was presented by Suzuki et al. in 1976. Instances of invasive thymoma with intravascular invasion of the SVC that extends into the RA have been rarely documented. It has been suggested that small thymomas located in the anterior mediastinum may infiltrate the brachiocephalic vein and subsequently proliferate along the venous flow into the SVC and down into the RA in a polypoid fashion [25, 27, 28]. In our case, the mediastinal mass was located in the anterior mediastinum, the classical site of thymoma, and showed morphology fully consistent with this diagnosis. The concomitant cardiac mass had identical features, supporting the interpretation of both tumors as thymoma. Because of this concordance, additional IHC was not performed. However, we acknowledge that IHC could have provided complementary confirmation, and its absence is a limitation of this study.

Primary cardiac lymphomas are also rare and mostly occur in immunodeficient persons. In the case of cardiac lymphomas, the RA and right ventricle are the most commonly involved areas [29, 30]. Histologically, cardiac lymphomas exhibit a range of B‐cell proliferations, including follicle center cell lymphomas, immunoblastic lymphomas, diffuse large cell lymphomas (the most common primary cardiac lymphoma), Burkitt lymphoma, and rare occurrences of T‐cell lymphoma, small lymphocytic lymphoma, and plasmacytic lymphoma. Immunocytochemical staining, cytogenetic analysis, and polymerase chain reaction (PCR) are used to confirm lymphoid lineage and identify the presence of a monoclonal population [30, 31].

Primary cardiac synovial sarcoma is an uncommon condition that presents a wide range of differential diagnoses, which may encompass mesothelioma, fibrosarcoma, leiomyosarcoma, undifferentiated sarcoma, and myxoma. The average age of patients diagnosed with cardiac synovial sarcoma is 32.5 years, spanning from 13 to 66 years. There is a distinct male predominance (male‐to‐female ratio of 3.5∶1) [32]. We report a case of primary cardiac synovial sarcoma originating from the RA. This tumor commonly develops in the pericardium or the right side of the heart, particularly within the RA. Macroscopically, cardiac synovial sarcomas often appear as polypoid masses with a smooth surface, displaying the same range of morphologic features as synovial sarcomas found in soft tissues (Figure 3). They may present in either a biphasic form (with both spindle cell and epithelial cell areas) or a monophasic form (containing only spindle cells) [33]. This sarcoma is a rare MT of soft tissue. Around 80% of cases are found in proximity to major joints or tendon tissues in the limbs. Unusual locations for the occurrence of SS include the head and neck, pleura, heart, kidneys, and prostate [34]. The diagnosis of primary cardiac synovial sarcoma in our case was based on typical morphology and a supportive IHC panel, which is considered sufficient in routine practice. Cytogenetic or molecular confirmation, such as detection of the SS18‐SSX fusion transcript, was not performed because of limited availability. This represents a limitation of the present study, although the combined morphologic and immunophenotypic findings strongly supported the diagnosis.

Primary undifferentiated pleomorphic sarcoma is an extremely rare disease and commonly occurs in the left atrium. This sarcoma is typically a poorly differentiated malignant mesenchymal tumor with fibroblastic or myofibroblastic characteristics. It is composed of atypical spindle cells exhibiting various levels of atypia, mitotic activity, necrosis, and nuclear polymorphism. Most of these tumors are found in the left atrium. The differential diagnosis includes cardiac myxoma and other soft tissue sarcomas. Cardiac myxomas are distinct in that they lack cellular atypia and frequent mitotic figures, and they express calretinin [31]. A definitive diagnosis of undifferentiated pleomorphic sarcoma is established by ruling out other malignancies through a panel of immunohistochemical markers [35]. The presentation of UPS may vary significantly, ranging from asymptomatic cases with incidental discoveries to severe manifestations such as heart failure, arrhythmias, or cardiac tamponade. Managing this condition poses considerable challenges. The recommended approach to treatment typically involves a multidisciplinary strategy that includes surgery, chemotherapy, and radiotherapy [36].

Finally, we acknowledge that the small sample size limits the strength and generalizability; however, this reflects the exceptional rarity of malignant cardiac tumors over the period. Also, a limitation of the present study is the small number of cases for each tumor type and incomplete follow‐up information in some instances. Another limitation of our study is the lack of detailed treatment outcomes and long‐term follow‐up data, which is common in rare case series. Therefore, while the findings highlight the importance of histopathologic confirmation in cardiac tumors, further multicenter studies are necessary to establish more specific diagnostic and therapeutic recommendations tailored to each MT subtype.

## 5. Conclusion

Most cardiac tumors are benign, while malignant ones are rare but life‐threatening. Some malignant cardiac tumors are metastases from other organs. Therefore, there is a wide variety of cardiac tumor types, which increases the difficulty of diagnosis. This report presents five cases of rare malignant cardiac tumors, providing valuable insights into their pathological characteristics. These cases contribute significantly to the growing body of knowledge, offering critical data to refine diagnostic techniques and optimize treatment protocols. Additionally, understanding the epidemiology and presentation of these tumors can aid in earlier detection and more effective management strategies.

## Author Contributions

Mahshid Hesami: conceptualization, supervision, and writing—original draft. Kambiz Mozaffari: writing—review and editing. Behnaz Jahanbin: resources and writing—review and editing.

## Funding

No funding was received for this manuscript.

## Disclosure

No identifiable information has been disclosed, and due care has been taken to protect patient privacy, particularly given the small sample size.

## Ethics Statement

Ethical approval was granted by the Ethics Committees of Rajaie Cardiovascular Medical and Research Center, Iran University of Medical Sciences, Tehran, Iran (IR.RHC.REC.1403.103). Written informed consent was obtained from the participants. We confirm that all patient data included in this study has been fully anonymized in accordance with the journal′s policies and institutional ethical guidelines.

## Conflicts of Interest

The authors declare no conflicts of interest.

## Data Availability

Data sharing is not applicable to this article as no datasets were generated or analyzed during the current study.
